# Hypophosphatemia Regulates Molecular Mechanisms of Circadian Rhythm

**DOI:** 10.1038/s41598-018-31830-7

**Published:** 2018-09-13

**Authors:** Takashi Noguchi, Amira I. Hussein, Nina Horowitz, Deven Carroll, Adam C. Gower, Serkalem Demissie, Louis C. Gerstenfeld

**Affiliations:** 1 Orthopaedic Research Laboratory, Department of Orthopaedic Surgery, Boston University School of Medicine, Boston, USA; 2 Clinical and Translational Science Institute, Boston University School of Medicine, Boston, USA; 3 Department of Biostatistics, Boston University School of Public Health, Boston, MA 02118 USA

## Abstract

Transcriptomic analysis showed that the central circadian pathway genes had significantly altered expression in fracture calluses from mice fed a low phosphate diet. This led us to hypothesize that phosphate deficiency altered the circadian cycle in peripheral tissues. Analysis of the expression of the central clock genes over a 24–36 hour period in multiple peripheral tissues including fracture callus, proximal tibia growth plate and cardiac tissues after 12 days on a low phosphate diet showed higher levels of gene expression in the hypophosphatemia groups (p < 0.001) and a 3 to 6 hour elongation of the circadian cycle. A comparative analysis of the callus tissue transcriptome genes that were differentially regulated by hypophosphatemia with published data for the genes in bone that are diurnally regulated identified 1879 genes with overlapping differential regulation, which were shown by ontology assessment to be associated with oxidative metabolism and apoptosis. Network analysis of the central circadian pathway genes linked their expression to the up regulated expression of the histone methyltransferase gene EZH2, a gene that when mutated in both humans and mice controls overall skeletal growth. These data suggest that phosphate is an essential metabolite that controls circadian function in both skeletal and non skeletal peripheral tissues and associates its levels with the overall oxidative metabolism and skeletal growth of animals.

## Introduction

In vertebrates circadian rhythms are mediated by a central set of clock genes: CLOCK, NPAS2, BMAL1, BMAL2, PER1, PER2, PER3, CRY1, and CRY2^1,2^. These genes are regulated in a diurnal manner by a negative feedback loop that is controlled both at the transcriptional and post-translational levels^2^. The clock genes provide an internal time-keeping system within individual cells that controls both systemic and tissue specific circadian biological responses. In mammals a central clock regulates systemic responses and is found within the superchiasmatic nucleus (SCN) and is controlled by the daily light-dark cycle through a subset of retinal ganglion neurons that express melanopsin that is transmitted directly to the SCN via the retinohypothalamic tract. The central clock then entrains the peripheral clocks at the local organ and tissue levels through both endocrine and neuronal signals^1^.

Peripheral clock systems are independently sensitive to daily levels of physical activity, local metabolic needs and the overall metabolic state of the animal^3^. Several studies have shown that restricted feeding regimens^4,5^ have the ability to shift the oscillatory phase within peripheral clocks of specific tissues, while having no effect on the oscillatory patterns in the SCN. Numerous studies have also shown that scheduled exercise during the daytime leads to phase advancement of circadian clock in multiple peripheral tissues in rodents^6,7^. Finally, while exercise accelerates clock gene cycles in mouse skeletal muscle and lung tissues, it does not alter the SCN to a new light-dark cycle^8^. On the other hand, ablation of the SCN has no effect on the peripheral entrainment of clock genes to either restricted feeding or physical activity^9^.

Skeletal tissues are one of the peripheral tissues that express a strong circadian clock and numerous studies have shown that bone metabolic functions are regulated in a circadian manner^10,11^. Bone tissues, like muscle and adipose tissue, also show entrainment of the clock genes in response to exercise, food intake, and metabolic state^12^. Growth plate tissues and chondrocytes within the growth plate, like bone tissues, also showed a strong circadian expression of the clock genes^13^ and PTH treatment was shown to directly regulate the circadian oscillation of the clock genes, particularly in the cartilage^14^. Subsequent studies from the same group of investigators showed that chondrocyte differentiation that occurred during the endochondral bone formation processes of fracture healing in mice showed a strong circadian oscillation of the clock genes and was also regulated by PTH treatment^15^.

Dietary and congenital hypophosphatemia during development causes rachitic disorders that are associated with the expansion of the epiphyseal growth plates of long bones, a failure in growth chondrocyte replacement with bone tissue, and leads to an overall retardation in growth. Mechanistic studies have shown that the expansion of the hypertrophic chondrocytes within the growth plate are related to decreased numbers of cells undergoing mitochondria-mediated apoptosis^16,17^. Within bone tissues hypophosphatemia causes osteomalacia and is associated with impaired mineralization of the extracellular matrix. Recent studies of acute phosphate restriction were shown to both increase marrow adiposity and impaired vascular tissue development within the marrow^18^. Acute phosphate restriction also leads to delayed bone healing after fracture and recapitulates many cellular and molecular effects that are seen in rachitic growth plates^19^. Studies of fracture healing under conditions of acute phosphate restriction further showed that skeletogenic stem cell differentiation was impaired, which was mechanistically related to decreased bone morphogenetic protein signaling within these cells^19^.

Our prior studies of endochondral bone formation that takes place during fracture healing in mice fed a low phosphate diet, showed similar effects on chondrocyte differentiation as seen within epiphyseal growth plates of growing mice^19^. Our studies examining the time course of the fracture healing transcriptome in control mice and those under conditions of dietary phosphate restriction identified that the circadian clock gene pathway was strongly regulated in response to hypophosphatemia. Given that PTH, the primary hormone that regulates systemic phosphate levels was shown to also regulate the central circadian clock in differentiating chondrocytes of both epiphyseal growth plates and during the endochondral phase of bone formation of fracture healing^14,15^, we hypothesized that levels of systemic phosphate itself might regulate the peripheral circadian clock genes in multiple tissues. The studies outline below test this hypothesis.

## Materials and Methods

### Animals and Husbandry

Animal research was conducted in conformity with all federal and USDA guidelines, under an approved Boston University, Institutional Animal Care and Use Committee protocol. Circadian rhythm studies were carried out using 8–10 week old male C57BL/B6J mice and Per2:Luciferase knock in mice^20^ in a C57BL/B6J background. Microarray studies were carried out using 8–10 week old male C57BL/B6J, A/J, and C3H/HeJ mice. Remaining studies were carried out using 8–10 week old male C57BL/B6J mice. All mice were obtained from Jackson Laboratories, Bar Harbor ME and were housed at Boston University Laboratory Animal Science Center under 12 h light dark cycle with free access to food and water. Mice were randomly divided into a normal diet group (controls) and phosphate restricted diet group (Pi) containing 0.06% phosphate compared to 0.4% in normal diet (Teklad 2018; 0.65% phosphorus). The phosphate restricted diet group (Pi) was fed the low phosphate diet starting two days before fracture and continued for 16 days after which, a normal diet was re-introduced. A hypophosphatemic state was confirmed by measurement of serum phosphate levels: after 12 days, control mice had a mean serum phosphate concentration of 10.92 ± 0.85 mg/dl, whereas mice on phosphate restricted diets were 6.91 ± 1.07 mg/dl (p < 0.001).

### Closed Fracture

Simple closed mid diaphyseal femoral fractures and their fixation was as previously described^21^ and were produced under general anesthesia with isoflurane. The fracture device as described by Marturano *et al*.^22^ was used to generate closed fractures by blunt trauma^22^.

### Tissue Harvest

Circadian rhythm studies were performed at day 10 post fracture. A 24 hour light dark cycle using Zeitgeber time (ZT) in which time 0 was defined when lights were turned on (7 AM) and ZT12 when lights were turned off (7 PM) was used. Three hour increments from ZT3 up ZT24 or ZT6 day 2 were assessed with n = 3 mice per time point (mRNA isolation) and n = 8 luciferase assay and serum. Heart, callus, and the proximal tibia tissues containing the growth plates and serum were collected.

### Serum analysis

Serum was obtained from whole blood obtained from cardiac punctures at the time of euthanasia. The concentration of serum phosphate was evaluated using Phosphate Colorimetric Kit (Sigma-Aldrich, IL, USA) according to manufacturer’s protocol. PTH and FGF23 ELISA assays were carried out at the Massachusetts General Hospital Center for Skeletal Research Cores Facilities Cell Signaling Assay Core as per posted protocols for the core facility (Immutopics 60–2305 and 60–6300 kits).

### Analysis of Cartilage and Bone by CECT and μCT

Calluses were scanned at a resolution of 12 µm/voxel (µCT40, Scanco Medical, Brüttisellen, Switzerland) before and after eight hours of incubation in a cationic contrast agent, CA4+^23^, which labels cartilaginous tissues (n = 10–12 per group). Cartilage volumes were determined as previously reported^24,25^ while mineralized tissue, total callus, mineralized callus volume fraction, and tissue mineral density (considering only mineralized tissue) were quantified^26^. Preexisting cortex and intramedullary volume were excluded from these calculations.

### RNA Isolation

RNA was extracted as previously described^19^ using Qiazol lysis reagent (Qiagen, Valencia, USA) and homogenized using Qiagen TissueLyser II (n = 6 per group). RNA concentrations were determined based on the 260/280/310 nm ratios of their optical densities using a DU530 UV vis spectrophotometer (Beckman, Coulter, CA).

### Real time PCR

Real-time quantitative PCR was performed as previously described^27^ using an Applied Biosystems 7300 PCR machine. The comparative C_T_ method (∆∆C_T_)^28^ was used for quantification with 18S RNA as the endogenous reference and ratios were set to either no fracture or ZT0. The primers and probes used in present study are as described in Supplemental Table 1.

### Microarray analysis

All procedures were performed at the Boston University Microarray and Sequencing Resource as described in the manufacturer’s instructions. Triplicate mRNA pools made from a randomized pooling of mRNAs isolated from N = 6 callus were used. 200 ng of RNA from each of the three mRNA pools was labeled with biotin using the Ambion WT Expression Kit (Life Technologies, Grand Island, NY) according to the manufacturer’s protocol, followed by the GeneChip WT Terminal Labeling and Controls Kit (Thermo Fisher, Waltham, MA). Labeled, fragmented DNA was hybridized to the Affymetrix Mouse Gene 1.0 ST Array for 18 hours in a GeneChip Hybridization oven 640 at 45 °C with rotation (60 rpm). Hybridized samples were washed and stained using an Affymetrix fluidics station 450, and after staining, microarrays were immediately scanned using an Affymetrix GeneArray Scanner 3000 7G Plus.

A total of 239 samples were processed and scanned separately in five batches, which were balanced for all experimental variables, and included a set of 17 samples that were present in all batches as an internal control. All 239 CEL files were normalized together to produce gene-level expression values using the implementation of the Robust Multi-Array Analysis (RMA) algorithm^29^ in the affy R package (version 1.36.1) and an Entrez Gene-specific probeset mapping (version 17.0.0) from the Molecular and Behavioral Neuroscience Institute (Brainarray) at the University of Michigan. Array quality was assessed by computing Relative Log Expression (RLE) and Normalized Unscaled Standard Error (NUSE) values using the affyPLM R package (version 1.34.0), and by re-normalizing the CEL files using Expression Console (build 1.3.0.187) to generate the Area Under the [Receiver Operating Characteristics] Curve (AUC) metric using positive and negative control probes. All samples had similar distributions of RLE and NUSE values, and AUC values greater than 0.85, indicating that all samples were of suitable quality for analysis.

Expression values were processed using the implementation of ComBat in the sva R package (version 3.4.0) to adjust for any technical effects with respect to microarray processing, and the ComBat-adjusted expression values of the 17 internal control samples were averaged across all five batches of microarrays. All microarray analyses were performed using the R environment for statistical computing (version 2.15.1). Raw CEL files and RMA-normalized, ComBat-adjusted data have been deposited in the Gene Expression Omnibus (Series GSE99580). Differences between gene expression in the Pi and the control groups were examined using Analysis of covariance (ANCOVA) with time and strain as covaariates (SAS 9.4, Inc., Cary, NC). Correction for multiple hypothesis testing was accomplished using Storey’s positive false discovery rate (pFDR)^30^.

### Analysis of Periodicity of Per2 in Callus Organ Culture

A base formulation of DMEM 1x Glutamax (Gibco) media minus inorganic phosphate with 1x B-27 supplement was custom purchased from Gibco MD, USA and reconstituted using MilliQ deionized water (Millipore, Medford, MA). Media was made 10 mM HEPES (Lonza, Basel, Switzerland), 1% Penicillin/Streptomycin (Gibco). For control conditions, mono and di basic phosphate was added back to a final concentration of 1.00 mM, while for PI deficient media, phosphate had a final concentration of 0.25 mM. Calluses from 10 day post fracture were aseptically dissected from mice in both groups (n = 8 in each group). Each callus was split in half using a bone cutter. Half callus tissues were placed into 35 mm culture dish with 2.4 ml of the culture medium. At the time of assay tissues was transferred to 96 well white opaque plate with the same medium during the measurement period and 200 μM Beetle Luciferin (Promega) was added at the time of each assay. Plates were read on a Synergy2 plate reader which was continuously run twice for each bioluminescence measurement. After each measurement, the tissues were returned to their 35 mm dish. Measurements were performed every three hours from first measurement (defined as 0 hour) to the 13th time point (36 hours). Values were calculated by detrending method^31^ to reveal the phase shift between the control and Pi condition. The detrended data was analyzed by JTK_CYCLE software, version 3.1.

### Statistical and Informatics analysis

All qPCR expression values were normalized to the lowest expression value across all time points in the control group. A gene was considered to have an oscillatory pattern across a 24 hour period if the mean expression was found to be significantly different across timepoints by a one-way ANOVA (analysis of variance) and expression was significantly higher at one or more consecutive time points compared to the lowest value using a post-hoc test (Dunnett’s test). In addition, to examine the overall effect of diet on gene expression, the values were compared between diets and among time points using a two-way analysis of variance (ANOVA). The periodicity of Per2 was evaluated using the Wilcoxon test. The concentration of serum phosphate was compared using an unpaired t-test. Statistical analysis was performed using JMP Pro ver12 (SAS Institute, Cary, NC). All results were expressed as the mean with standard deviation. A p value less than 0.05 was considered as a significant difference.

Ingenuity Pathway Analysis (IPA) software (http://www.ingenuity.com/) was used to identify biological and disease functions and known canonical pathways that were significantly dysregulated with respect to phosphate restriction (diet) (FDR *q* < 0.05). The parameters for the network analysis were set to a maximum of 25 networks with 70 molecules per network.

## Results

### Transcriptomic Effect of Acute Phosphate Restriction on Circadian Functions in Fracture Callus Tissues

**Figure 1:**
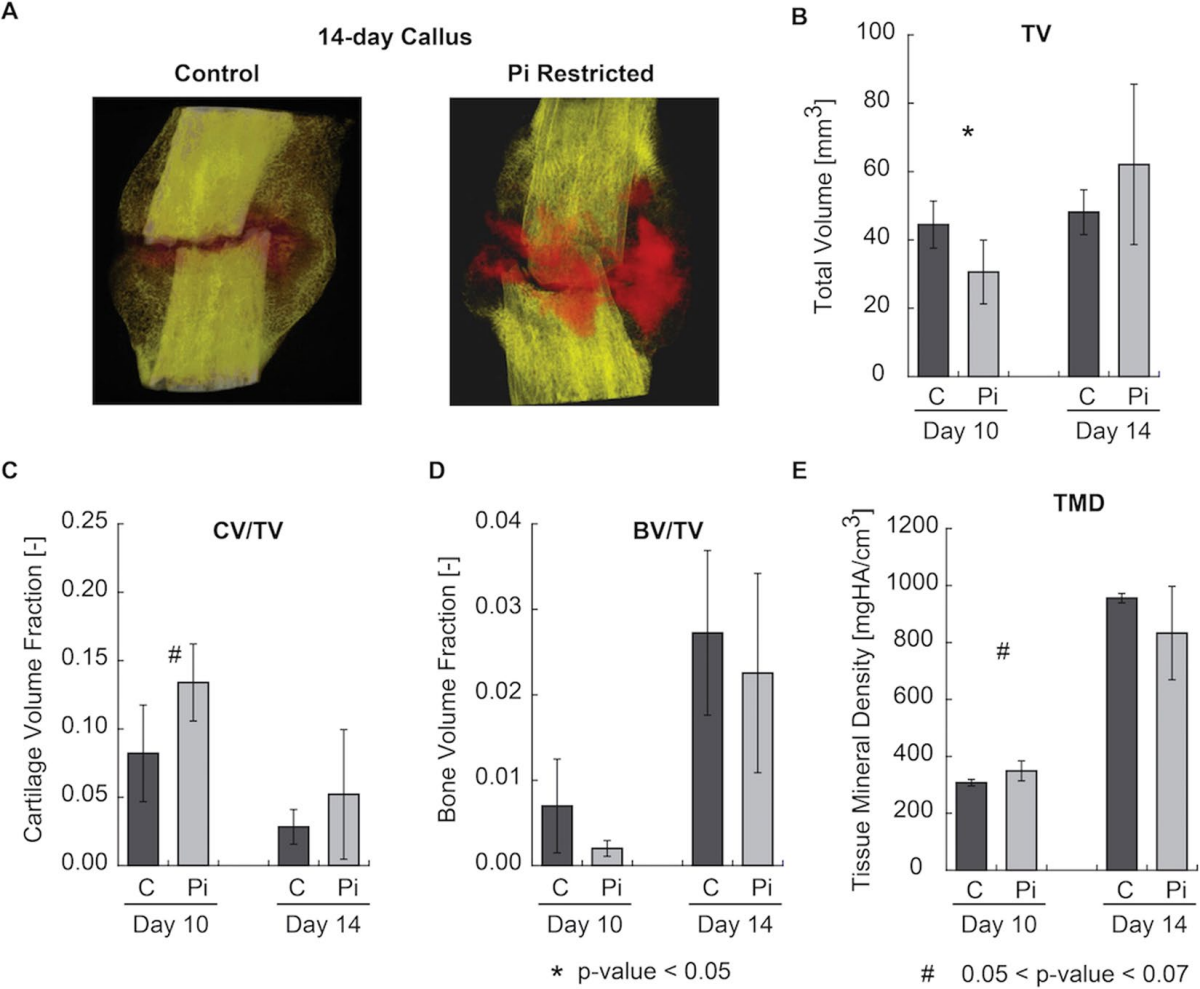
Organ-level effects of hypophosphatemia on endochondral bone formation during fracture healing: (**A**) Renderings of representative calluses (cortex included) at days 14 imaged using contrast-enhanced micro-computed tomography, with mineralized tissue rendered in yellow/white and cartilage in red; (**B**) Total callus volume; (**C**) Cartilage volume fraction (the ratio of cartilage volume to total callus volume); (**D**) Bone volume fraction; and (**E**) Total mineral density of callus tissues. *A significant t-test p-value comparing Pi and Control expression levels at each time point (p < 0.05). ^#^A trend in t-test p-value comparing Pi and Control expression levels at each time point (0.05 < p < 0.07).

The organ-level effects of hypophosphatemia on endochondral bone formation during fracture healing were examined by micro CT analysis (Fig. 1A). This analysis showed that initiation of hypophosphatemia two days prior to fracture and maintenance on a low phosphate diet through the first fourteen days of healing led to an overall increase in the total cartilage volume fraction as determined by contrast-enhanced analysis of the calluses (Fig. 1B,C). Although the volumes of the callus tissues were initially lower in the phosphate deficient group, by fourteen days there was no difference relative to the control group. As was expected, overall total mineral density (TMD) and bone volume ratio (BV/TV) trended lower in the hypophosphatemic groups (Fig. 1D,E).

**Figure 2:**
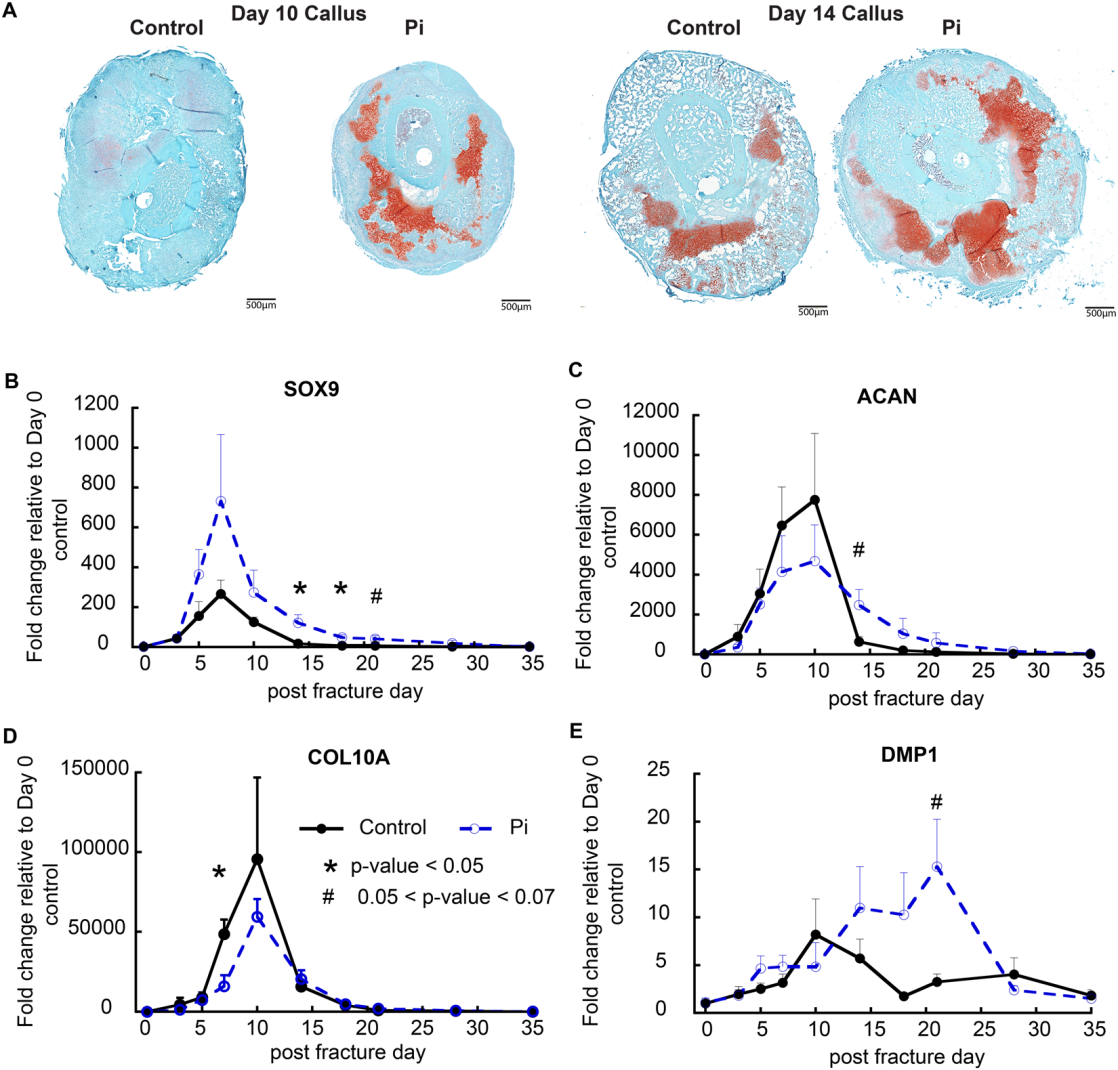
Effects of hypophosphatemia on the progression of cartilage and bone cell differentiation and identification of altered circadian function during fracture healing. (**A**) Histological analyses of the effects of hypophosphatemia on post-operative day 10 and day 14 callus tissues. Callus sections in the mid-transverse plane of control and hypophosphatemic (Pi) mice are shown. Callus tissues from hypophosphatemic mice displayed large regions of residual cartilage and large regions of lesser differentiated cells tissue abutting the fracture. (**B**–**E**) Expression of cartilage and bone-related genes as denoted in the figure: Error bars represent ±1 standard error of the mean of the 2–3 repeat analyses performed on six samples per group. ^*^A significant t-test p-value comparing Pi and Control expression levels at each time point (p < 0.05). ^#^A trend in t-test p-value comparing Pi and Control expression levels at each time point (0.05 < p < 0.07).

Qualitative histological examination (Fig. 2A) confirmed the conclusions that had been drawn from the micro CT studies, showing both greater and more prolonged levels of cartilage tissues at day 10. Selective qRT-PCR was used to further assess cartilage and bone cell differentiation in the two groups of mice (Fig. 2B–G) across the period in which fracture calluses tissues were forming. These results showed that osteochondro-lineage progenitors and early committed chondrocytes which express Sox9 were elevated in the hypophosphatemic mice. In contrast proliferative matrix forming cells as assessed by aggrecan expression were lower and the presence of these cells was more prolonged. Messenger RNA expression associated with hypertrophic cells (Col10a1) (Fig. 2B–D) and secondary bone cell differentiation (Dmp1) was delayed and lower in hypophosphatemic group up to day 14 however, when the animals were placed back on a normal phosphate diet after day 14 expression of the DMP1 was greatly elevated and the period of initial bone formation was prolonged and greatly elevated (Fig. 2E).

A comparative temporal analysis of the callus tissue transcriptomes across the experimental time course was next carried out on RNA isolated from three strains of control and hypophosphatemic mice to gain a more comprehensive and mechanistic understanding of the effects of hypophosphatemia on post-natal endochondral bone formation. A total of n = 7188 genes were identified by ANCOVA with diet effect FDR q value ≤ 0.05. Interestingly, the core transcription factors controlling circadian function (Per2 and Per3) were among the top regulated molecules (Table 1), which were upregulated relative to control groups of RNA during phosphate deficiency (days 3–14) and subsequently reverted to control levels following a switch to normal phosphate diet (days 18–35).

**Table 1:** Top molecules regulated by hypophosphatemia.

Symbol	Entrez Gene Name	Entrez Gene ID	FDR (q value)	Fold Change
Ndufa2	NADH:ubiquinone oxidoreductase subunit A2	17991	3.12E-10	−1.15
Ppp1r11	protein phosphatase 1 regulatory inhibitor subunit 11	76497	4.49E-10	−1.13
Bmal1	aryl hydrocarbon receptor nuclear translocator like (ARNTL)	11865	1.86E-09	−1.257
Per2	period circadian regulator 2	18627	4.61E-09	1.211
Per3	period circadian regulator 3	18628	8.42E-09	1.205
Mir-18	microRNA 17	387135	9.67E-09	−1.212
Fkbp4	FK506 binding protein 4	14228	1.44E-08	−1.115
Atp5e	ATP synthase, H+ transporting, mitochondrial F1 complex, epsilon subunit	67126	2.32E-08	−1.164
Mrpl53	mitochondrial ribosomal protein L53	68499	2.32E-08	−1.088
Pla2g7	phospholipase A2 group VII	27226	2.32E-08	1.151

A set of 7188 genes that were significantly associated with diet (ANCOVA with diet effect FDR q < 0.05) was used to perform functional annotation in Ingenuity Pathway Analysis. The fold change for the Pi group relative to the control group across all timepoints is listed.

**Figure 3:**
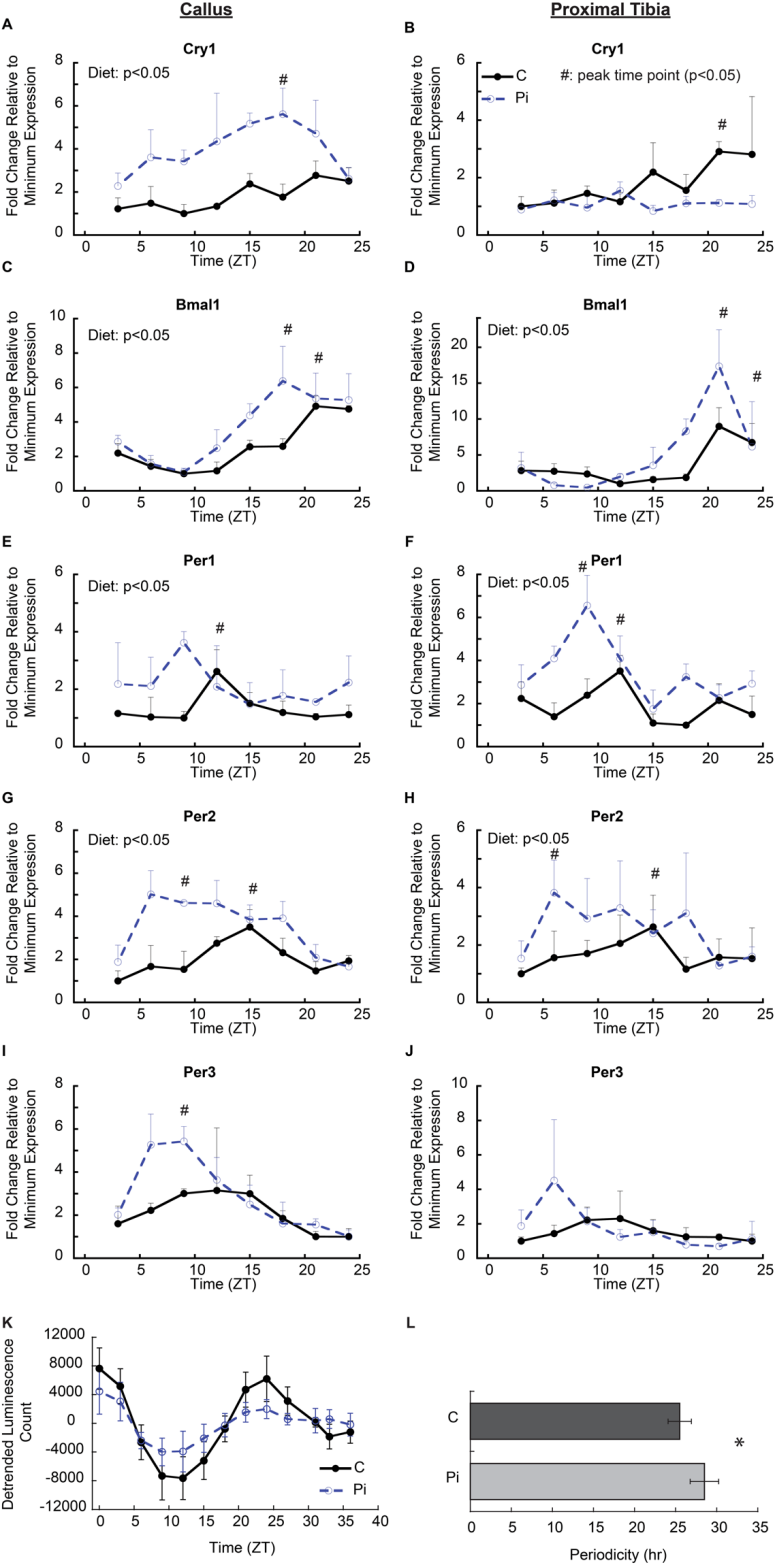
Alteration of circadian regulatory gene expression by hypophosphatemia in callus and growth plate tissues. (**A**–**J**) mRNA expression of central circadian clock genes in callus and growth plate tissue isolated over a 24 hour period 12 days after the mice were placed on a hypophosphatemic diet. Tissues were harvested every three hours from mice in both groups (n = 3, in each group by time points). Error bars represent ±1 standard error of the mean of the 2–3 repeat analyses performed on six samples per group. ^#^Indicates peak time point (p < 0.05); A significant diet effect in two-way ANOVA (p < 0.05) is indicated in the upper left of the figure. (**K**) Analysis of Per2-driven bioluminescence in callus tissues of control (C) and hypophosphatemic (Pi) animals when cultured *ex vivo* over a 39 hour period in medium containing 1.0 mM or 0.25 mM phosphate, respectively. (**L**) Bar graphs of detrended data calculating the periodicity of Per2 in control (25.12 ± 1.45 hours) and Pi (27.86 ± 2.45 hours) (p = 0.023). n = 8 in each group.

Since these static mRNA assessments only inferred that hypophosphatemia affected circadian function, a direct measurement of the central molecular mechanisms of circadian regulation was carried out in both callus and the proximal tibia growth plate tissues over a twenty-four-hour period in both control and hypophosphatemic mice (Fig. 3A–J). In both callus and growth plate tissues under control conditions, peak levels of Per1 expression were seen at ZT12 and Per2 and Per3 peak levels were at ZT15. In contrast, Bmal1 peaked at ZT21, countercyclical to the Period genes. These data reflect the well-defined circadian rhythmic pattern for the central clock genes in these tissues. Tissues from the hypophosphatemic mice showed significantly higher levels of expression for most of the central circadian genes with an observed phase shift of peak expression to ZT6-ZT12 in Per2, ZT6-ZT9 in Per3, and ZT18-ZT24 in Bmal1. A defined elevation and shift in the expression of Cry1 gene expression was not observed in the growth plate relative to its expression in callus tissue, which showed a broad peak of expression from ZT 15–21 in both control and hypophosphatemic tissues while levels in the hypophosphatemic tissues were much higher throughout the 24-hour period that it was measured.

Since the phosphate deficiency *in vivo* might have numerous systemic mediators that could affect the central circadian mechanism, the effect of phosphate deficiency on the central circadian function was directly assessed *in vitro* using callus tissues harvested from mice containing the Per2 promoter driving the luciferase gene. In these *in vitro* studies the callus tissues were placed in tissue culture media containing either normal (1.0 mM) or low (0.25 mM) phosphate concentrations. The bioluminescent signal showed well defined sinusoidal curves in both groups of callus tissues (Fig. 3K). In these studies the amplitude of the group placed in low phosphate media was slightly smaller than that of the control group; however, it should be noted that the overall callus volume at this time point was ~30% less than that of control mice, which would account for the lower levels of overall expression of the luciferase indicator. The periodicity calculated from the data was 25.12 ± 1.45 hr in the control group, and 27.86 ± 2.45 hr in the Pi group (*p* = 0.02) (Fig. 3L). These *ex vivo* organ culture data indicate that phosphate levels had a direct effect on the central circadian mechanism in the callus tissue, which are consistent with our twenty-four-hour mRNA profiles in which hypophosphatemia moved the first peak in the rhythm approximately 3–5 hours earlier however in the *ex vivo* context we did not observe a phase shift.

### Hypophosphatemia Has Systemic Effects on Circadian Functions of Non Skeletal Tissues

**Figure 4:**
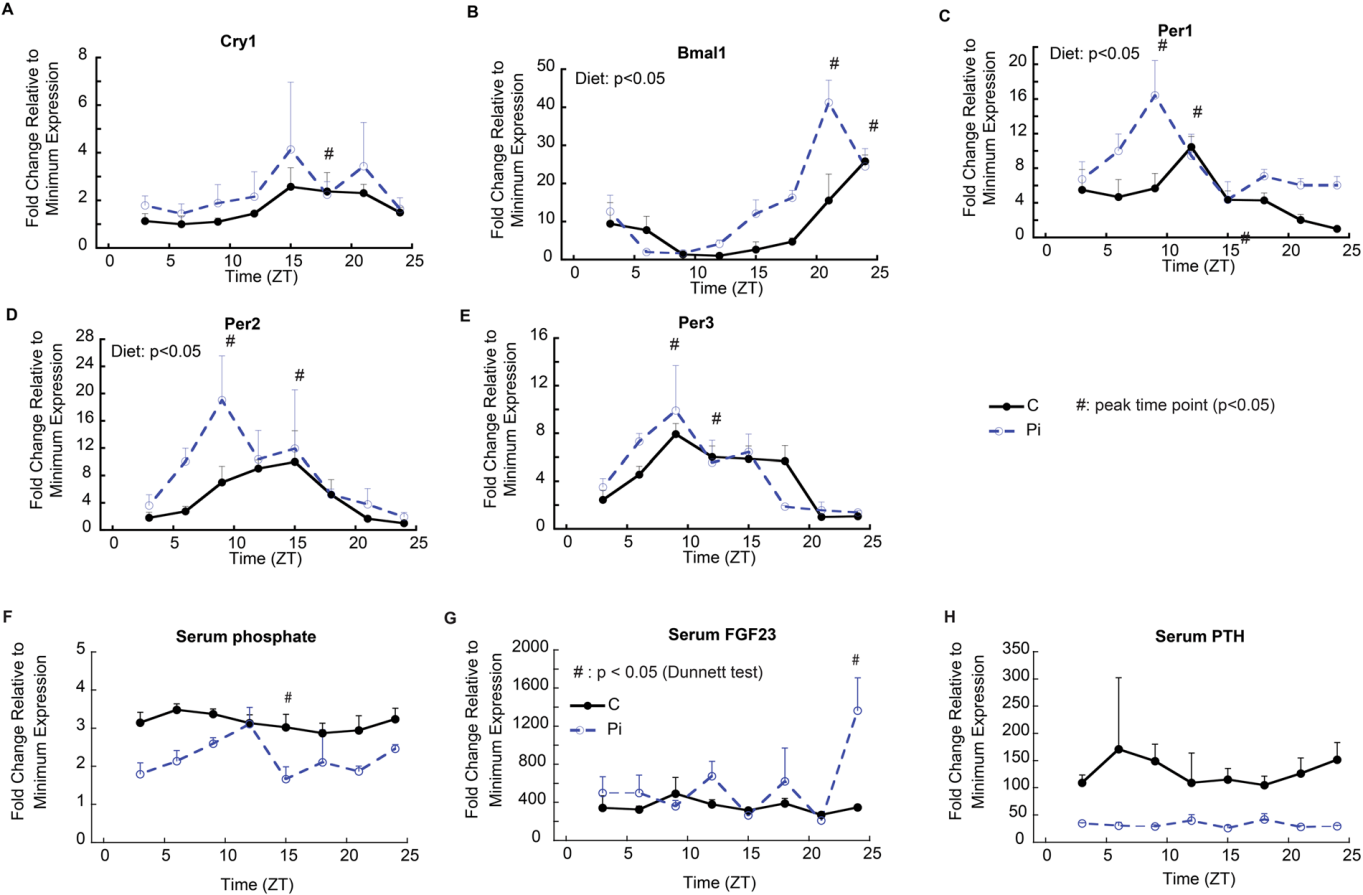
Alteration of circadian regulatory gene expression by hypophosphatemia has systemic effects on other peripheral issues and on systemic regulation of mineral metabolism. (**A**–**E**) mRNA expression of central circadian clock genes in cardiac tissue isolated over a 24 hour period 12 days after the mice were placed on a hypophosphatemic diet. Tissues were harvested every three hours from mice in both groups (n = 3, in each group by time points). Error bars represent ±1 standard error of the mean of the 2–3 repeat analyses performed on six samples per group. ^#^Indicates peak time point (Dunnett’s p > 0.05, versus lowest value across all time poitns) in each dietary group, denoted by color. ^*^Significant diet effect in two-way ANOVA (p < 0.05). (**F**–**H**) Effect of hypophosphatemia on serum diurnal phosphate levels and the expression of the two known systemic regulators of phosphate mineral metabolism. ^#^Dunnett’s test p-value < 0.05.

While varying metabolic and developmental effects of phosphate metabolism have been extensively documented in skeletal tissues, its effects on other tissues have not. Since numerous peripheral tissues have been shown to have strong circadian rhythms that respond to alterations in metabolic conditions, we examined one of these tissues (heart)^32^ to determine if hypophosphatemia affected its circadian function. Heart tissues from control animals showed peak levels for Per1, 2 and 3 at ZT12, ZT15 and between ZT9-ZT18, respectively, while Bmal1 had a peak level at ZT24 and Cry1 had a broad peak between ZT15 and ZT24. In contrast, hypophosphatemic mice showed significantly higher levels and the phase moved to earlier time points: ZT9 for Per1, ZT9 for Per2 and ZT6-15 for Per3. Bmal1 was similarly shifted forward to ZT21. Therefore, as in cartilaginous tissues, the oscillating pattern showed a phase shift in heart tissue in response to hypophosphatemia (Fig. 4A–E).

Phosphate, PTH and FGF23 levels were next examined as systemic indices of circadian alterations, since they are known to exhibit a circadian pattern in their serum concentrations^33,34^. The effect of hypophosphatemia on their circadian pattern in serum is presented in Fig. 4F–H. Clear alterations in peak levels and concentrations of the profiles for PTH and phosphate were seen however none of these differences were statistically different; however, FGF23 levels, while showing significant elevation in the serum of the hypophosphatemic mice, did not show a clear diurnal pattern in our experiments.

### Linkage of Circadian Function to the Temporal Spatial Mechanisms that Control Growth

**Figure 5:**
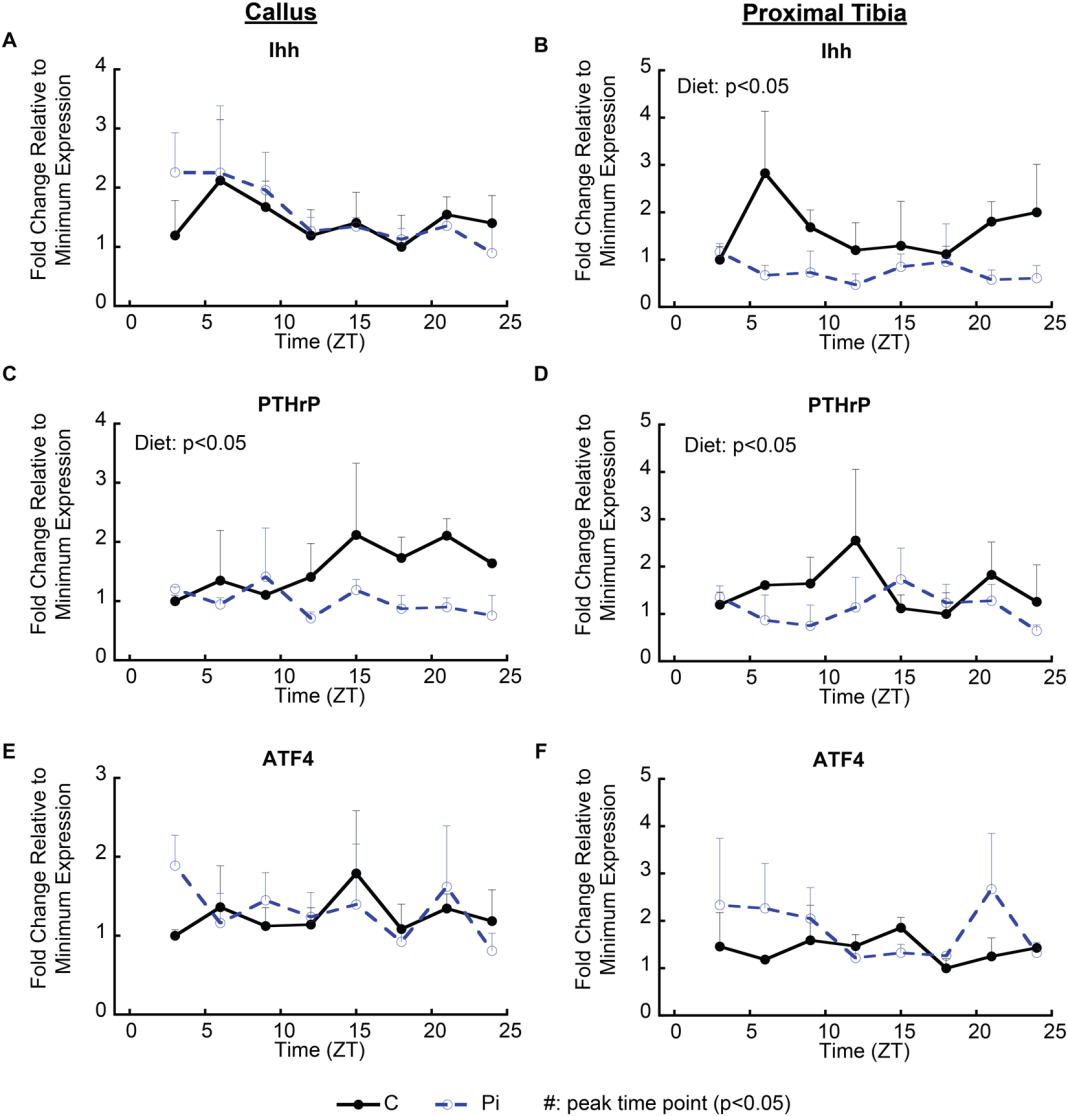
Characterization of regulators of controlling temporal callus growth in response to hypophosphatemia. (**A**,**B**) mRNA expression of Ihh, a major morphogenetic factor controlling the growth cartilage feedback loop that regulates chondrocyte differentiation. (**C**–**F**) mRNA expression of PTHrP and Atf4, primary transcription factors controlling cartilage proliferation and apoptosis. Tissues were harvested every three hours from mice in both groups (n = 3, in each group by time points). Error bars represent ±1 standard error of the mean of the 2–3 repeat analyses performed on six samples per group. ^*^Significant diet effect in two-way ANOVA (p < 0.05). Symbol was attached beside the value point when significant higher value among the time points was detected (post-hoc test) in each dietary group and per individual time.

In order to assess if the known negative feedback loop between PTHrP and Ihh that controls cartilage tissue hypertrophic differentiation and growth of chondrocytes^35^ is regulated in a circadian manner, we examined the expression of these two genes. We also included Atf4 as another possible target since microarray analysis showed that Atf4 expression was decreased by hypophosphatemia across time (diet FDR *q* = 0.0001). The potential role of this gene in circadian regulation was suggested since it is known to regulate chondrocyte programmed death^36^ and terminal chondrocyte apoptosis has been shown to be regulated by hypophosphatemia^16^. While hypophosphatemia significantly decreased the expression of PTHrP, and Ihh had a strong peak expression in both callus and growth plate tissues at ZT4 hours, there was no discernable diurnal shift in the pattern of their expression in the hypophosphatemic group when these data were examined for a diurnal trend (Fig. 5A–D). There was also no significant difference in Atf4 expression, either across the 24 hour period or between the groups, in either of the two tissues (Fig. 5E,F).

Since circadian regulation did not overtly overlay the PTHrP and Ihh feedback loop, we then hypothesized that other molecular mechanism(s) must be affected by hypophosphatemia that control the temporal aspects of growth. In order to identify what these mechanisms are, we intersected the 7188 genes that were significantly associated with diet (FDR *q* < 0.05) in our transcriptomic study with a published set of genes under circadian regulation in mouse calvaria^11^ (the complete list of these genes was downloaded from the “Research” link on the Stem Cell Biology website at (http://labs.pbrc.edu/stemcell), producing a list of 1879 genes (Summarized in Supplemental data Table 2). A breakdown of the primary biological functions and canonical pathways that were regulated in both a circadian manner and in response to phosphate deficiency showed that mitochondrial function inclusive of oxidative metabolism and canonical regulatory pathways associated with apoptotic signaling showed the greatest statistical significance (Table 2).

**Table 2:** A breakdown of the primary biological functions and canonical pathways that were regulated in both a circadian manner and in response to phosphate deficiency.

Canonical Pathways	−log10(p-value)	Diseases or Functions Annotation	−log10(p-value)
Mitochondrial Dysfunction	13.2	cell death	13.88
Oxidative Phosphorylation	11.6	necrosis	13.64
Protein Ubiquitination Pathway	10.2	organismal death	12.01
NRF2-mediated Oxidative Stress Response	4.62	morbidity or mortality	11.72
Glutaryl-CoA Degradation	4.61	apoptosis	10.49
PTEN Signaling	4.43	cell death of connective tissue cells	8.39
Aldosterone Signaling in Epithelial Cells	4.22	size of embryo	7.68
Apoptosis Signaling	4.17	cell death of fibroblast cell lines	6.69
Huntington’s Disease Signaling	4.04	processing of RNA	6.23
Hypoxia Signaling in the Cardiovascular System	4.01	necrosis of muscle	6.12
PI3K/AKT Signaling	3.75	Growth Failure	6.01
STAT3 Pathway	3.35	morphology of body cavity	5.68
Rac Signaling	3.28	morphology of heart	5.66
TCA Cycle II (Eukaryotic)	3.18	neuronal cell death	5.66
IGF-1 Signaling	3.16	omphalocele	5.59
B Cell Receptor Signaling	3.14	morphology of cardiovascular system	5.46
Ephrin Receptor Signaling	3.07	arrest in G2 phase of fibroblast cell lines	5.23
Regulation of eIF4 and p70S6K Signaling	3.04	interphase	5.23
NF-κB Signaling	3.03	proliferation of connective tissue cells	5.03
Glucocorticoid Receptor Signaling	2.99	anemia	5.03

A set of 1,879 genes that were significantly associated with diet and also reported as having a circadian pattern of regulation in mouse calvaria was used to perform functional annotation in Ingenuity Pathway Analysis.

**Figure 6:**
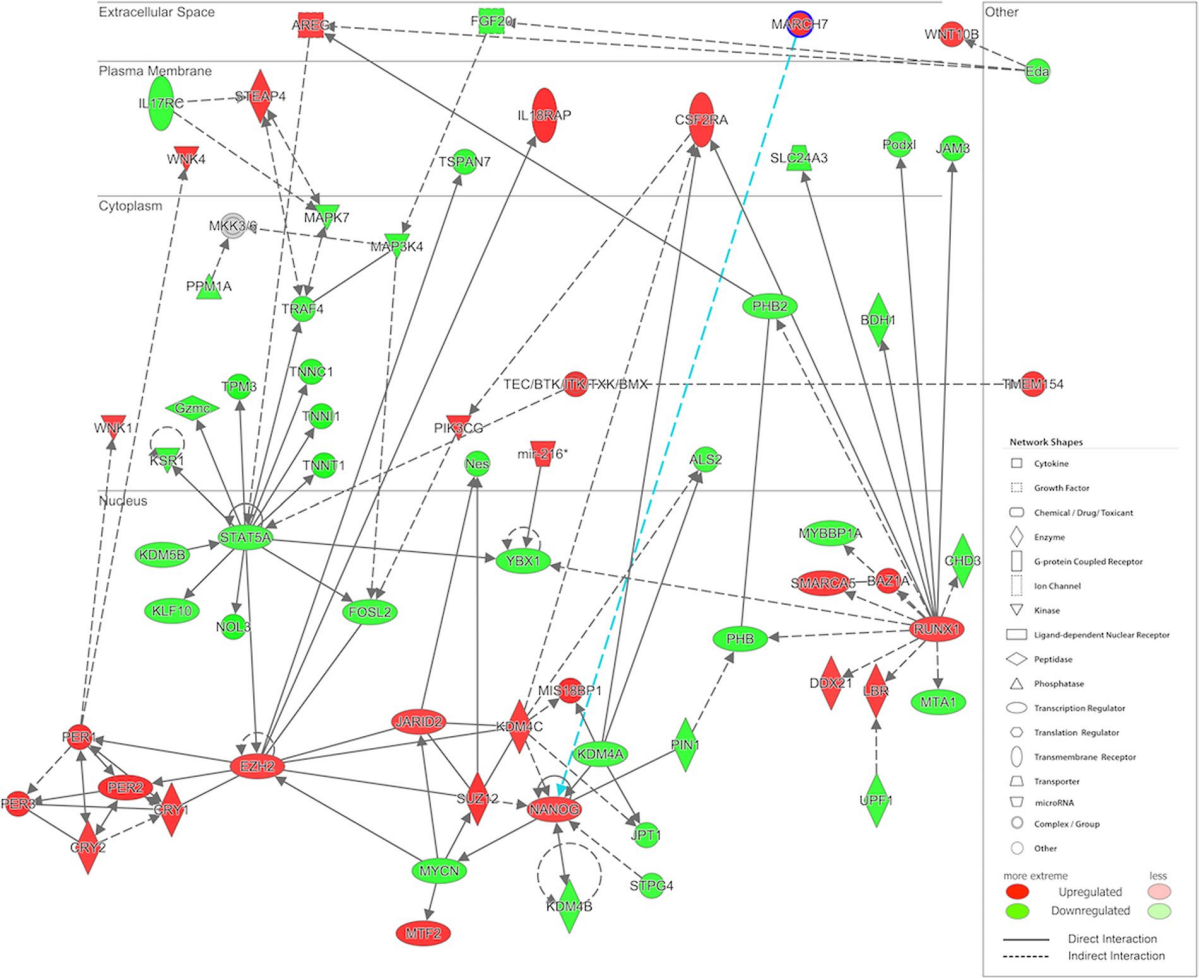
A network predicted from genes significantly associated with the diet contains the central group of genes associated with circadian function. Network was generated by Ingenuity Pathway Analysis software. Colors represent the fold change of the Pi group relative to the control group across all timepoints, and are scaled so that red and green indicate genes that are up- or down-regulated, respectively, in Pi compared to the control group, with color intensity indicating the degree of change.

To gain further insight into the molecular circuity through which the central core of circadian of factors regulated growth, a network was constructed using the 7188 genes that were significantly associated with diet (FDR *q* < 0.05) in our transcriptomic study (Fig. 6). It contains a number of key transcriptional regulators that have been shown to both regulate embryonic stem cell commitment to mesoderm^37^ and mesenchymal stem cell maintenance during post-natal bone repair (e.g., Nanog)^38,39^ and initial progression of mesenchymal stem cells to the chondrocyte lineage (e.g., Runx1)^40,41^. This network also contains the gene encoding the histone methyltransferase EZH2, which has recently been identified as an epigenetic regulator of growth chondrocyte proliferation and hypertrophy^42^ and has been shown to be the casual gene of skeletal overgrowth in Weaver Syndrome^43,44^.

## Discussion

### The Relationship between Circadian Function and the Molecular Regulators of Mineral Metabolism

The data presented here suggests that phosphate mineral metabolism controls peripheral circadian functions. Consistent with our studies that lead us to this conclusion are data showing that PTH, which leads to both a hyperphosphatemic state and increases the rate of chondrocyte differentiation, causes an inverse effect of shortening of the clock genes’ diurnal expression cycle in growth plates, fracture callus tissues and murine chondrocytes and chondroprogenitor cells^14,15,45,46^. The interplay between the regulatory mechanisms of mineral metabolism and circadian functions is also supported by multiple research studies in osteoblasts. PTH was shown to strongly downregulate the E4BP4 gene a basic leucine zipper transcription factor that is known to be a primary downstream regulator of circadian function in osteoblasts^47^ and in more recent data α1-adrenergic receptor regulation in osteoblast cells, was shown to independently regulate cell growth and function in osteoblasts through its effect on the circadian expression of E4BP4 gene^48^. In other studies that directly link circadian function to the mechanism that control mineral metabolism, the global deletion of murine Bmal1 was shown to lead to a low bone mass due to an increase in bone resorption. This observation was mechanistically refined though the targeted deletion of Bmal1 in osteoblasts, which alone supported increased osteoclastogenesis facilitated by increased induction of RANKL through 1–25-dihydroxyvitamin D3 [1,25(OH)2D3] mediated signaling^47,49^. Finally, in a different study, it was shown that the core circadian gene Clock regulated bone formation via transcriptional control of the 1,2,5(OH)2D3 receptor PDIA3, which directly modulated osteogenic apoptosis^48,50^.

The temporal mechanism(s) that overlay the negative PTHrP and Ihh feedback loop, which provides the molecular basis for the temporal/spatial period of growth chondrocyte differentiation and longitudinal long bone growth, has not been established. Although PTHrP showed decreased expression in hypophosphatemic groups from both callus and growth plate tissues, it lacked a definable diurnal peak in our studies. On the other hand, while Ihh showed a distinct diurnal peak consistent with prior findings^49,51^, its expression in both tissues failed to show the same response. These finding then suggest that the temporal period of this negative feedback cycle is not directly controlled through a circadian mechanism that regulates either PTHrP or Ihh gene expression.

### Role of EZH2 Expression in the Mediating the Effects of Hypophosphatemia

The histone methyltransferase Ezh2, which is part of a network of genes that is differentially regulated by hypophosphatemia, and which interacts with the central elements of the circadian of regulatory machinery, is a strong potential candidate mechanism for providing temporal control of the overall rate of growth. In this regard, a previous study has shown that EZH2 binding and di- and trimethylation of H3K27 on both the Per1 and Per2 promoters and its targeted silencing led to a loss of circadian function of mouse cells^50,52^. Furthermore, EZH2 has been genetically linked to skeletal longitudinal growth through various mutations associated with a number of rare overgrowth disorders in humans, characterized by prenatal and/or postnatal overgrowth, accelerated osseous maturation, characteristic craniofacial features, intellectual disability, and limb anomalies^43,44^. In two recent reports, the targeted knockout of Ezh2 in chondrocytes^42^ or in Prx1-expressing mesenchymal stem cell populations^51^ was shown to lead to limb length shortening and overall runting of animal growth. Both of these studies identified that loss of EZH2 led to Cdkn2c- and Cdkn2a-mediated inhibition of skeletogenic cell proliferation^42,51^. The ablation of EZH2 specifically within nestin-expressing marrow MSCs led to a more restricted osteoporotic phenotype in trabecular bone of only mature animals. These authors specifically attributed the development of osteoporosis to the specific decrease in the number of MSCs or osteoprogenitors in mature bone^52,53^.

The identification of Runx1 and Nanog, two known transcription factors found in skeletal stem cells^39–41,53,54^, in the same network as both the circadian genes and Ezh2 suggests that hypophosphatemia controls growth through the regulation of the resting zone skeletogenic stem cells that give rise to chondrocytes of the growth plate as well as decreasing the rate of their terminal differentiation and programmed death. This interpretation is consistent with studies that have shown that polycomb-mediated repression of the differentiation of both embryonic and multiple postnatal stem cell lineages through EZH2 activity maintains them in a stem cell state and delays their terminal differentiation by controlling their apoptosis^55,56^. The functional relationship of circadian function and apoptosis and senescence^57,58^ has also been noted in number of studies as well as in studies of embryonic stem cells^56,59^.

Finally, these finding are consistent with prior mechanistic studies that have shown that phosphate levels regulate apoptosis of hypertrophic chondrocytes through activation of the caspase-9-mediated mitochondrial pathways and the MEK1/2-ERK1/2 signaling pathways^16,17^. The comparison of our transcriptomic changes to those that are regulated in a circadian manner showed that the most predominant canonical regulatory pathways controlled by both phosphate and in a circadian manner were those associated with programmed cell death and metabolism. In this regard the relationship circadian function and metabolic activity has been extensively validated^60^.

In summary, our results suggest that phosphate levels, through their regulation of circadian function, control the rate of skeletogenic stem cell differentiation, initially slowing the rate of chondrogenic lineage differentiation then preventing apoptotic exit of these cells. These data further suggest that phosphate controls overall skeletal growth through its regulation of intermediate metabolism. Thus our results suggest that phosphate levels regulate the rate of apoptosis throughout differentiation of skeletogenic cells from their stem cell state through their terminal differentiation.

## Electronic supplementary material

Supplementary Information
